# Nucleophilic Addition of Reactive Dyes on Amidoximated Acrylic Fabrics

**DOI:** 10.1155/2014/305930

**Published:** 2014-08-28

**Authors:** Reda M. El-Shishtawy, Manal M. El-Zawahry, Fatma Abdelghaffar, Nahed S. E. Ahmed

**Affiliations:** ^1^Chemistry Department, Faculty of Science, King Abdulaziz University, P.O. Box 80203, Jeddah 21589, Saudi Arabia; ^2^Dyeing, Printing and Textile Auxiliaries Department, Textile Research Division, National Research Center, Dokki, Giza 12622, Egypt

## Abstract

Seven reactive dyes judiciously selected based on chemical structures and fixation mechanisms were applied at 2% owf of shade on amidoximated acrylic fabrics. Amidoximated acrylic fabric has been obtained by a viable amidoximation process. The dyeability of these fabrics was evaluated with respect to the dye exhaustion, fixation, and colour strength under different conditions of temperature and dyeing time. Nucleophilic addition type reactive dyes show higher colour data compared to nucleophilic substitution ones. FTIR studies further implicate the binding of reactive dyes on these fabrics. A tentative mechanism is proposed to rationalize the high fixation yield obtained using nucleophilic addition type reactive dyes. Also, the levelling and fastness properties were evaluated for all dyes used. Excellent to good fastness and levelling properties were obtained for all samples irrespective of the dye used. The result of investigation offers a new method for a viable reactive dyeing of amidoximated acrylic fabrics.

## 1. Introduction

Acrylic fabric is a wool-like fabric with outstanding chemical and physical properties such as lightweight, soft, high strength, and good abrasion and insect resistance. These characteristics have led to its application in textile industries as a less expensive alternative to wool [1]. Acrylic fabric, which is a synthetic polymer made from a copolymer of acrylonitrile containing 1–15 mass % of several vinyl comonomers containing carboxylate or sulphonate groups, is conventionally dyed with cationic dyes [2, 3]. As acrylic fabrics are widely used in textile blends with natural fabrics such as wool and cotton, thus the viability of using different dyes for the colouration of acrylic fabrics would have a positive impact on textile industries. In this regard, reactive dyes and anionic dyes such as acid dyes are not usually used for acrylic colouration as these dyes suffer from being not substantive for the fabrics as a result of the repulsive effects that occur between the anionic groups present in the fabrics and those present in the dye molecules. For this purpose and in the interest of making acrylic fabrics less hydrophobic, anionic dyeable, a facile, safe, and efficient pretreatment method in which a partial conversion of nitrile groups present in the fabrics into amidoxime groups has been reported [4]. The amidoxime groups as the active dye sites have proven effectiveness in increasing the substantivity of acrylic fabrics toward anionic dyes at an acidic pH [4–7].

Amidoxime is effective α-nucleophile and its O-nucleophilic characteristics revealed a unique capability of reacting with an acylating agent in the basic, neutral, and acidic media [8, 9]. Additionally, an unusually fast nucleophilic addition of amidoximes to acetylene has been reported [10]. This outstanding nucleophilicity of amidoxime group prompted us to reveal the mechanism of reactive dye fixation on amidoximated acrylic fabrics. Reactive dye fixation proceeds by two main mechanisms; nucleophilic addition and nucleophilic substitution and this mechanism depend on the dye type and the textile substrate [11, 12].

In continuation of our interest in anionic colouration of acrylic fabrics and stemmed by the results of one-bath reactive dyeing of amidoximated wool/acrylics in which a remarkable high reactive dye fixation value was obtained at acidic pH [7], it was of interest to investigate the dyeing characteristics of amidoximated acrylic fabrics using different types of reactive dyes. The purpose of this work was to explore the viability of reactive dye fixation on amidoximated acrylic fabrics for the first time and to determine which type of fabric reactive dyes is suitable for such fabrics (i.e., nucleophilic addition or nucleophilic substitution type reactive dyes). Pretreatment of acrylic fabrics and its impact on tensile strength is presented. The pretreated fabrics are then subjected for colouration with different reactive dyes under different conditions and the fastness properties of the dyed samples are then evaluated.

## 2. Experimental

### 2.1. Materials

#### 2.1.1. Acrylic Fabrics

The acrylic fabrics used in this study was a 1/1 woven acrylic (40.65 × 40.65 threads/inch for both weft and warp) with 0.36 g/cm^3^ density, supplied by Misr El-Mehalla Co., Egypt. The fabric was soaped with 2 g/L nonionic detergent (Hostapal CV, from Clariant-Egypt) at 60°C for 30 min, thoroughly rinsed and air dried.

#### 2.1.2. Dyestuffs and Chemicals

The dyes used in this work were CI Reactive Blue 19 (RB19), CI Reactive Orange 16 (RO16), CI Reactive Violet 5 (RV5), CI Reactive Red 84 (RR84), CI Reactive Blue 171 (RB171), CI Reactive Red 4 (RR4), and CI Reactive Red 195 (RR195). These dyes were kindly supplied by Ciba-Egypt and were used as received. All other chemical reagents were of laboratory grade.

### 2.2. Pretreatment and Dyeing

#### 2.2.1. Pretreatment

Following our previously described method [4], a known mass of acrylic fabric was pretreated with hydroxylamine hydrochloride (0–14 g/L) using aqueous solutions of ammonium acetate (20 g/L) at a liquor-to-goods ratio of 50 : 1 at 85°C for 60 min. The pretreated samples were thoroughly rinsed with water and air dried.

#### 2.2.2. Dyeing with Reactive Dyes

The pretreated acrylic samples were introduced into a dyebath of a liquor ratio 40 : 1 at 40°C of pHs 2.5 using McIlvaine buffer solutions [13, 14] and the temperature was raised to the desired temperature (60–100°C) over 20 min, and then the dyeing was continued at this temperature and under shaking for different time intervals (10–120 min). The dyed samples were removed from the dye pot, rinsed in cold water and soaped with 5 g/L nonionic detergent at a liquor ratio of 50 : 1 at 60°C for 30 min, rinsed with water and air-dried, and hereinafter called “wash-off 1.”

McIlvaine buffer solution was used to control the pH of the dyebaths at pH 2.5 during the dyeing process. This pH was obtained by mixing 3.42 mL of 0.2 M disodium hydrogen phosphate with 36.58 mL of 0.1 M citric acid to prepare 40 mL buffer solution. pH 2.5 was chosen according to our previous report in which higher reactive dye fixation was obtained on amidoximated wool/acrylic blend [7].

### 2.3. Measurements and Analyses

#### 2.3.1. Nitrogen Percentage

The percentage of nitrogen of the blank and pretreated wool/acrylic fabrics was determined by the Kjeldahl method [15]. The average values of three determinations were tabulated.

#### 2.3.2. FTIR Spectroscopy

Fourier transform infrared (FTIR) spectra were recorded on a Nexus 670 FTIR Spectrometer, Nicolet Company, USA, using potassium bromide disks. A total of 32 scans for each sample were taken with a resolution of 4 cm^−1^, with a range of 4000–400 cm^−1^.

#### 2.3.3. Tensile Strength

The warp tensile strength was determined for blank and pretreated wool/acrylic fabrics according to the ASTM strip test [16].

#### 2.3.4. Colour Measurements

The relative colour strength of dyed fabrics expressed as *K*/*S*, where *K* and *S* are the absorption and scattering coefficients, respectively, was measured by the light reflectance technique using the Kubelka-Munk equation (1) [17]:

(1) $$\begin{array}{c} \frac{K}{S}=\frac{{\left(1-R\right)}^{2}}{2R}. \end{array}$$

The reflectance (*R*) and the levelling properties of dyed samples were measured using a spectrophotometer (Hunter Lab Ultra Scan PRO Spectrophotometer (USA)) interfaced with a personal computer. In order to achieve correct readings, the spectrophotometer was set to exclude any specular components. Five readings per sample of one layer (moving the sample in between) were averaged.

The levelling properties of dyed fabrics using 2% owf dye applied at the selected dyeing conditions for all dyes were assessed by measuring the colour differences within each sample at five separate points and the average colour difference (Δ*E*) between these points was determined [18–20].

#### 2.3.5. Dye Exhaustion and Overall Fixation

The extent of dye exhaustion was determined spectrophotometrically. The absorbance of each dyebath solution before and after dyeing process was measured using 1 cm quartz cells housed in a Shimadzu UV-2401PC UV/visible spectrophotometer at the *λ*_max⁡_ of each dye. The percentage dyebath exhaustion (%*E*) was calculated using (2), where *A*_0_ and *A*_1_ are the absorbance of the dyebath before and after dyeing, respectively,

(2) $$\begin{array}{c} \%E=\frac{A_{0}-A_{1}}{A_{0}}\times 100. \end{array}$$

The extent of reactive dye fixation (%*F*) was determined by a method used by several researchers [5, 7, 21, 22] using (3) where (*K*/*S*)_1_ and (*K*/*S*)_2_ represent the colour strength of the dyeing after wash-off 1 and wash-off 2 (the washed sample by wash-off 1 was further washed with a solution containing 5 g/L nonionic detergent and 2 g/L sodium bicarbonate at a liquor ratio of 50 : 1, at the boil for 30 min, rinsed with water and air dried). This method assumes, at least at the concentration of dyes employed, that *K*/*S* values are proportional to concentration of dye on fabric. It is worth mentioning that the fixation value was estimated based on a soaping technique owing to the sensitivity of the pretreated acrylic fabrics to DMF and/or pyridine solution. From both %*E* and %*F* values, the overall percentage fixation, %*T*, was evaluated from

(3) %F=K/S1K/S2×100,

(4) %T=%E×%F100.

#### 2.3.6. Fastness Testing

Fastness testing for the dyed samples was tested according to ISO standard methods. The specific tests were ISO 105-X12 (1987), colour fastness to rubbing; ISO 105-C02 (1989), colour fastness to washing; and ISO 105-E04 (1989), colour fastness to perspiration.

## 3. Results and Discussion

**Figure 1:**
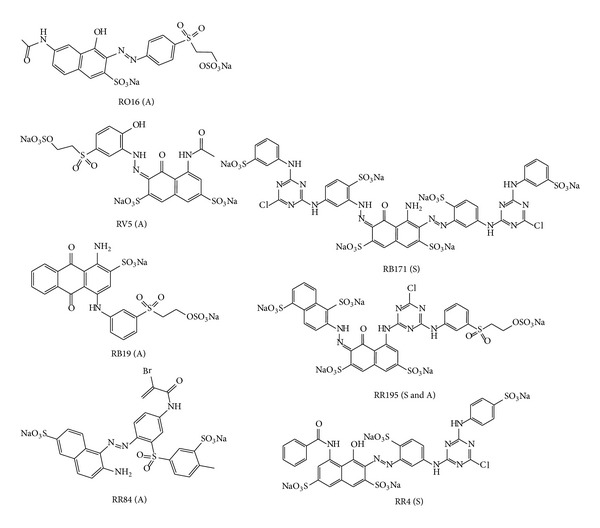
Nucleophilic substitution (S) and nucleophilic addition (A) type reactive dyes.

The unique reactivity of α-nucleophile in amidoxime group and the high uptake of anionic dye in an acid medium due to the presence of this group in the amidoximated acrylic fabrics [4–7] has prompted us to study the viability of reactive dye fixation in an acid medium using different types with different structures so as to find out which mechanism of fixation would be suitable for this type of substrate. For this purpose, amidoximation of acrylic fabrics was made following our previously reported method [4]. The amidoximated acrylic fabrics were then dyed using seven judiciously selected reactive dyes (Figure 1): four reactive dyes based on nucleophilic addition, one reactive dye based on nucleophilic substitution, and two bifunctional reactive dyes, one homobifunctional and the second heterobifunctional reactive dyes. It is needless to mention that the blank acrylic fabrics were nonsubstantive to all dyes used and the fabrics were colourless after being dyed under the appropriate conditions.

### 3.1. Amidoximation of Acrylic Fabrics

#### 3.1.1. Effect of Hydroxylamine Hydrochloride Concentration and Tensile Strength

Table 1 shows the effect of hydroxylamine hydrochloride concentration on the nitrogen content, tensile strength, and elongation at break of amidoximated acrylic fabrics. It is clear that nitrogen content increases as the concentration of hydroxylamine hydrochloride is increased. This result reveals the viability of amidoximation of acrylic fabrics, as the presence of amidoxime groups in the fabric would increase its nitrogen content relative to the blank one (compare nitrile group that has one nitrogen atom and amidoxime group that has two nitrogen atoms). It has been reported that amidoximation of acrylic and wool/acrylic fabrics resulted in lowering the crystallinity of the fabrics, as evidenced by X-ray data [3, 4]. Therefore, it is anticipated that the presence of amidoxime groups in the fabrics would render the fabrics more hydrophilic and less crystalline. It is known that the crystallinity of textile fabrics is correlated with the tensile strength for most textile fabrics [5]. Thus, lowering the crystalline phase and increasing the amorphous one of the amidoximated acrylic fabrics as a result of amidoximation would result in decreasing the tensile strength with increasing the concentration of hydroxylamine hydrochloride, as clearly observed in Table 1. Elongation at break, on the other hand, increases going from the blank to the amidoximated samples up to 10 g/L hydroxylamine hydrochloride above which the elongation starts to decrease. This result reflects the presence of microvoids present in the amidoximated fabrics due to the formation of amidoxime groups; however, increasing the concentration of hydroxylamine hydrochloride above 10 g/L would lead to an excessive amidoximation that produces less flexible fabrics as indicated by a lower value of elongation at break if compared with the blank sample. On the other hand, the tensile strength of amidoximated fabrics decreases more using concentration of hydroxylamine hydrochloride above 10 g/L. This result is in accordance with our previously reported results of acid dyeing of amidoximated acrylic fabrics, in which amidoximated acrylic fabrics with concentration of hydroxylamine hydrochloride above 10 g/L revealed lower dyeability owing to the increased compactness of the pretreated fabrics [4]. Hereinafter, the selected amidoximated fabrics are the one pretreated with 10 g/L hydroxylamine hydrochloride. Although this viable approach of amidoximation is a facile pretreatment process using water and nonvolatile soluble salt (hydroxylamine hydrochloride), yet caution has to be taken during the pretreatment owing to the toxicity of hydroxylamine hydrochloride as reported in the MSDS (http://www.sciencelab.com/msds.php?msdsId=9927192).

### 3.2. Dyeing with Reactive Dyes

#### 3.2.1. Effect of Dyeing Temperature

**Figure 2:**
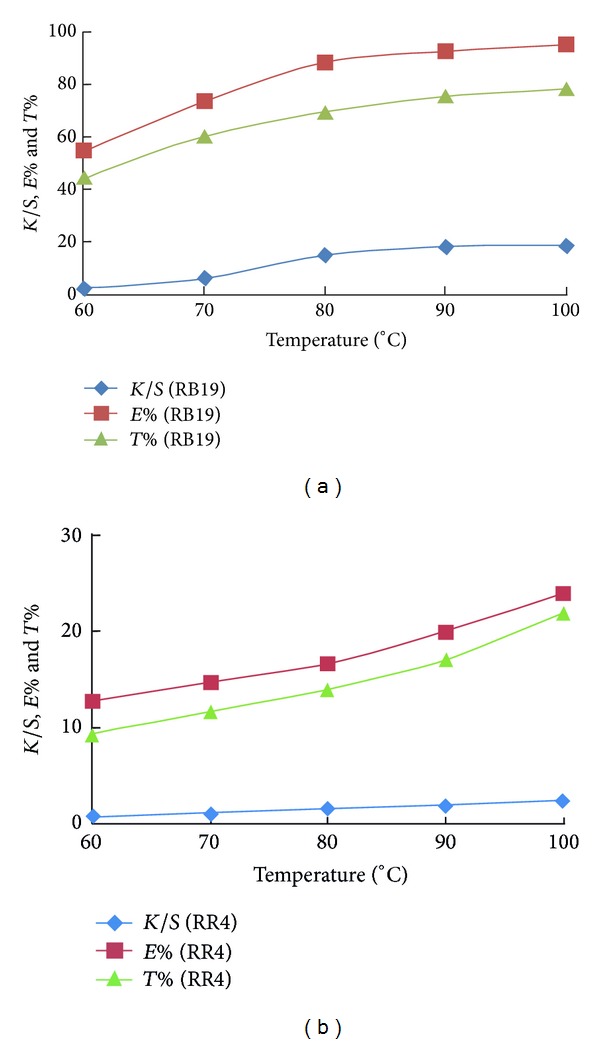
Effect of temperature on the exhaustion, total fixation, and colour strength of CI reactive blue 19 (A) and CI reactive red 4 (S) on amidoximated acrylic fabrics; dyeing conditions: shade 2% owf, liquor ratio 40 : 1, pH 2.5, for 60 min.

**Figure 3:**
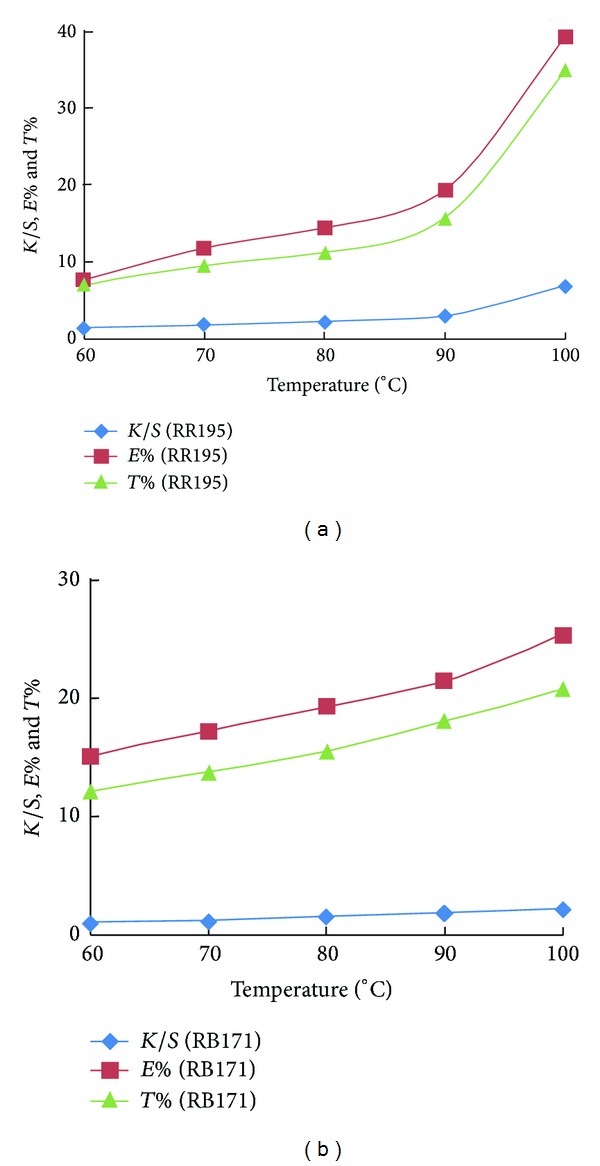
Effect of temperature on the exhaustion, total fixation and colour strength of CI Reactive Red 195 (heterobifunctional reactive dye) and CI Reactive Blue 171 (homobifunctional reactive dye) on amidoximated acrylic fabrics; dyeing conditions: shade 2% owf, liquor ratio 40 : 1, pH 2.5, for 60 min.

**Figure 4:**
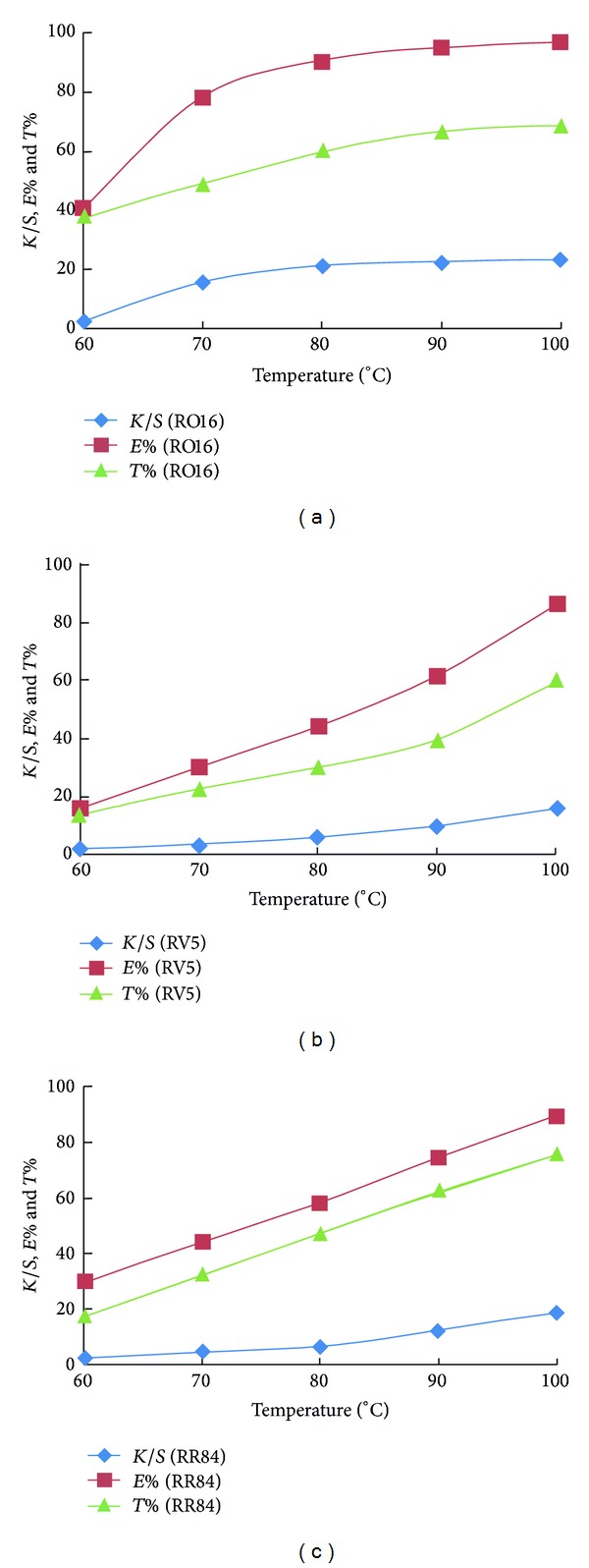
Effect of temperature on the exhaustion, total fixation, and colour strength of CI Reactive Orange 16 (A), CI Reactive Violet 5 (A), and CI Reactive Red 84 (A) on amidoximated acrylic fabrics; dyeing conditions: shade 2% owf, liquor ratio 40 : 1, pH 2.5, for 60 min.

Recently, the realization of one bath union shade dyeing of pretreated wool/acrylic fabrics was made using McIlvaine buffer solutions at pH 2.5 and in a dyebath of 40 : 1 liquor ratio [7]. These dyeing conditions of pH and liquor ratio were applied in the coloration of amidoximated acrylic fabrics using different reactive dyes and at different temperature and time so as to find out which mechanism of dyeing is suitable for such type of acrylic polymer. The effect of dyeing temperature on the dyeability of amidoximated acrylic fabrics with different reactive dyes is shown in Figures 2–4. It can be seen that the colour data represented as the colour strength, exhaustion, and total fixation values increase with the dyeing temperature. It is generally accepted that the dyeability enhancement upon heating is ought to the fabric swelling and, hence, better dye diffusion. In this context, the presence of amidoxime groups in the amidoximated acrylic fabrics as the active dye sites would facilitate dye uptake. It is worth noting the difference in the dyeability shown in Figures 2 and 3 in relation to the dye type.  Figure 2 compares the dyeability of amidoximated acrylic fabrics between RB19 that contains sulphatoethylsulphone (SES) group as a nucleophilic addition type reactive dye and RR4 that contains monochlorotriazine (MCT) group as a nucleophilic substitution type reactive dye. It is clear from the figure that the colour data obtained by nucleophilic addition are far better than those obtained by nucleophilic substitution. The result confirms that the selected pH was suitable for dye fixation using nucleophilic addition type reactive dyes but not for nucleophilic substation type ones. It seems that such low acidic pH even though it is good for dye exhaustion, it is not good for dye fixation using nucleophilic substation type reactive dyes owing to acid hydrolysis.

On the other hand, Figure 3 compares the dyeability of amidoximated acrylic fabrics between RR195 that contains SES group and MCT group (nucleophilic addition and nucleophilic substitution type reactive dye) and RB171 that contains two MCT groups (nucleophilic substitution type reactive dye). As expected, hetero bifunctional reactive dye resulted in a better colour data than those obtained by homobifunctional reactive dye owing to the presence of SES reactive site. However, the overall data of heterobifunctional reactive dye is not as high as of monofunctional nucleophilic addition type reactive dyes, which could be attributed to the lower affinity of RR195 due to its molecular size. This result confirms further the suitability of nucleophilic addition type reactive dyes for obtaining viable dyeing characteristics on amidoximated acrylic fabrics. However, the colour data obtained by RR195 was inferior to those obtained by RB19 and RR84. This result may be attributed to the molecular size effect and its impact on the dyeability. The smaller molecular size of RB19 and RR84 compared with RR195 (see Figure 1) may help further dye diffusion and thus better dye exhaustion.

Additionally, the effect of temperature on the dyeability of amidoximated acrylic fabrics was investigated using another two SES type reactive dyes, namely, RO16 and RV5 and RR84 that contains α-bromoacrylamide group as a nucleophilic addition type reactive dye. The results shown in Figure 4 support further the above findings of the good colour data obtained using nucleophilic addition type reactive dyes.

#### 3.2.2. Effect of Dyeing Time

**Figure 5:**
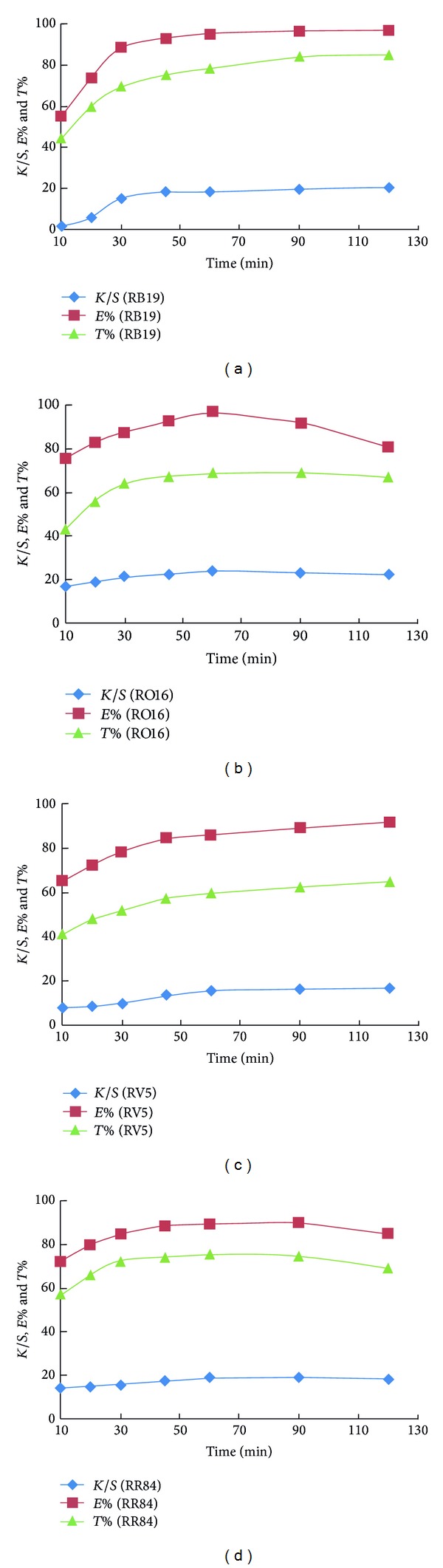
Colour strength, exhaustion, and total fixation of amidoximated acrylic fabrics using nucleophilic addition type reactive dyes of different structures as a function of time; dyeing conditions: shade 2% owf, liquor ratio 40 : 1, pH 2.5, at 100°C.

Figure 5 shows the effect of dyeing time on the colour strength, exhaustion, and total fixation of RB19, RO16, RV5, and RR84 on amidoximated acrylic fabrics. As indicated, the colour data increase with the dyeing time up to 60 min above which either a plateau is reached as in the case of RV5 and RB19 or a little decline in the dyeability takes place. It is known that the dyeing process proceeds via surface adsorption of dye molecules, sorption, diffusion, and dye-fabric fixation. This process reaches an equilibrium at certain time at which the sorption and desorption of the dye molecules are equal; however, prolonged dyeing time at such high temperature (100°C) would facilitate dye desorption as indicated in the values of colour strength and exhaustion values. The desorbed dye at such conditions could be attributed to the noncovalently fixed dyes and/or the hydrolyzed ones.

#### 3.2.3. Total Fixation of Reactive Dyes

**Figure 6:**
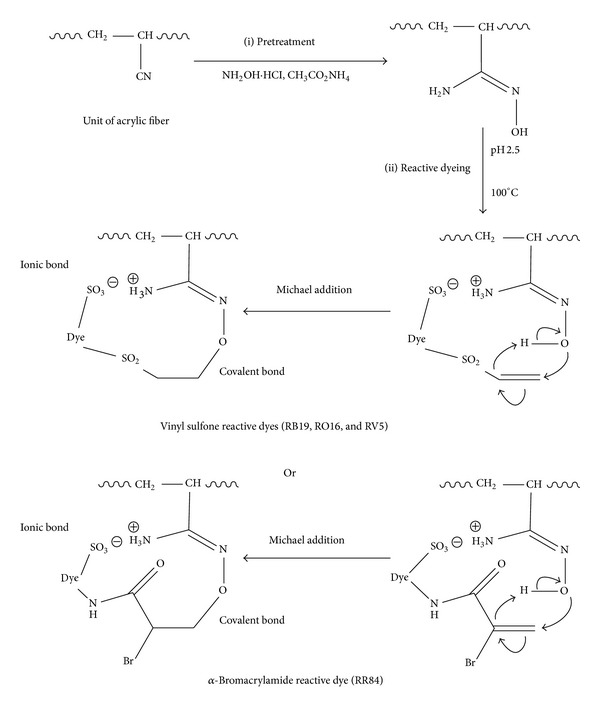
Fixation mechanism of reactive dyes on amidoximated acrylic fibres.

The remarkable high fixation values for RB19, RO16, RV5, and RR84 at such low acidic pH are attributed to the presence of amidoxime groups in the amidoximated acrylic fabrics. It is known that amidoxime group is an α-nucleophile type [8–10] in which O-nucleophile is the reaction site. Also, this acidic pH would favour the enhancement of dye exhaustion by virtue of ionic bond formation between the sulfonate groups present in the dye molecules and the protonated amino groups present in the amidoximated acrylic fabrics. This dye fixation mechanism can be schematized in Figure 6. Thus, SES containing reactive dyes (RB19, RO16, and RV5) get activated at high temperature to produce the Michael reactive form (vinyl sulfone) in which, upon being in close proximity with O-nucleophile present in the amidoximated fabrics, a covalent bond takes place via Michael addition mechanism. Also, Michael addition mechanism takes place between α-bromoacrylamide reactive dye (RR84) with the amidoximated acrylic fabrics.

#### 3.2.4. FTIR Analysis

FTIR spectra were made to further confirm the changes in the characteristic peaks of amidoxime groups present in the amidoximated acrylic fabrics after being dyed with reactive dyes. As elaborated above, only nucleophilic addition type reactive dyes are the one suitable for such type of fabrics and therefore three dyes of them are selected for FTIR analysis. The selected dyes are RB19, RO16, and RR84. RV5 was not selected as it is similar to RO16.

**Figure 7:**
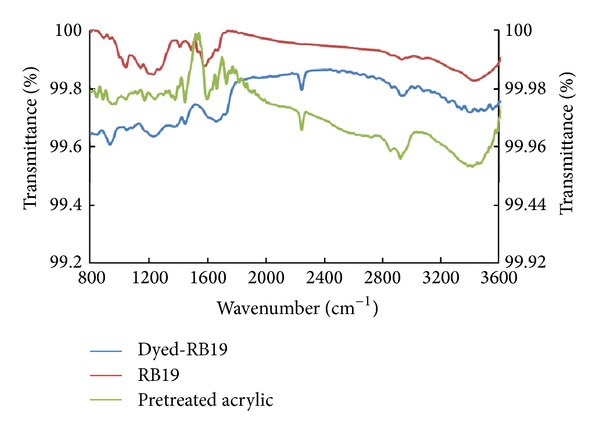
FTIR of amidoximated acrylic fibres, RB19, and amidoximated acrylic fabrics dyed with RB19; dyeing conditions: shade 2% owf, liquor ratio 40 : 1, pH 2.5, at 100°C for 60 min.

**Figure 8:**
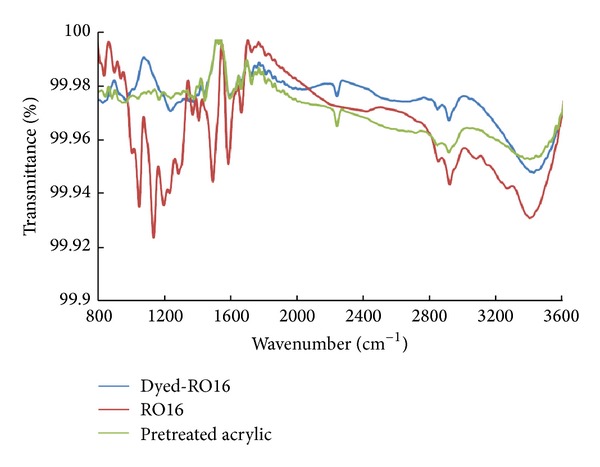
FTIR of amidoximated acrylic fibres, RO16, and amidoximated acrylic fabrics dyed with RO16; dyeing conditions: shade 2% owf, liquor ratio 40 : 1, pH 2.5, at 100°C for 60 min.

**Figure 9:**
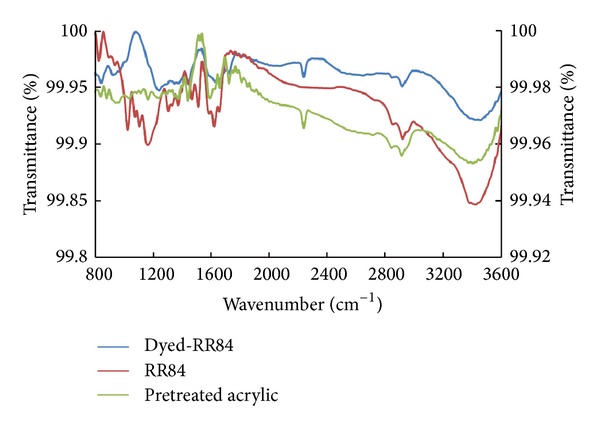
FTIR of amidoximated acrylic fibres, RR84, and amidoximated acrylic fabrics dyed with RR84; dyeing conditions: shade 2% owf, liquor ratio 40 : 1, pH 2.5, at 100°C for 60 min.

Figures 7, 8, and 9 show the FTIR spectra of amidoximated acrylic fabrics, the dye, and the fabric after being dyed with reactive dyes. The reaction of hydroxylamine hydrochloride with acrylic fabrics has partially converted some of the nitrile groups into amidoxime groups. Full FTIR data for the fabrics before and after pretreatment with hydroxylamine hydrochloride and has previously been reported by the present authors [4]. A characteristic band of nitrile groups at 2243 cm^−1^ is present on the surface of the fabric before and after dyeing. The characteristic C=N groups characterized by the band at 1594 cm^−1^. The broadband at 3150–3550 cm^−1^ can be attributed to H-bondings of NH_2_ and O–H in the amidoxime groups.

Tables 2, 3, and 4 summarize FTIR peaks for the amidoximated acrylic fabrics before and after dyeing with RB19, RO16, and RR84, respectively. Upon dyeing with reactive dyes, a shift was observed in the stretching band of N–O and C=N. This shift indicates the involvement of amidoxime groups in the fixation mechanism as shown in Figures 7–9. Also, the stretching band for OH and NH_2_ appeared with less intensity in the dyed fabrics using RB19 or RO16 as a consequence of losing the OH group in the fixation mechanism. However, using dye RR84, the stretching band before and after dyeing appeared with more intensity owing to the intensity contributed by the amino group present in the dye molecule. Furthermore, the presence of different bands in the dyed fabrics due to the stretching of sulfonic groups, carbonyl groups, bromide atoms, and the aromatic stretching vibrations with shift compared with those bands before dyeing indicated the binding of reactive dyes with the fabrics.

#### 3.2.5. The Levelling Properties

**Figure 10:**
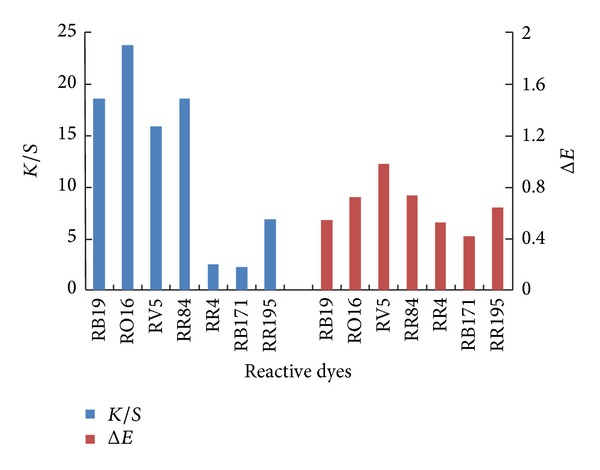
Comparative colour difference and colour strength of different reactive dyes on amidoximated acrylic fabrics; dyeing conditions: shade 2% owf, liquor ratio 40 : 1, pH 2.5, at 100°C for 60 min.

The levelling properties of dyed amidoximated acrylic fabrics are shown in Figure 10. It is clearly observed that the average colour differences (Δ*E*) of the dyed samples show good levelling properties in all cases (Δ*E* less than 1). It is worth noting the difference in the number of water solubilizing groups in the four reactive dyes shown in Figure 1. Both RB19 and RO16 have one sulfonic group, whereas both RR84 and RV5 have two sulfonic groups. Therefore, it is anticipated that RB19 and RO16 are less hydrophilic than RR84 and RV5 and as a consequence both RB19 and RO16 would reveal higher affinity to the amidoximated acrylic than those of RR84 and RV5. Indeed the *E*% and *T*% values of RB19 and RO16 are higher than those of RR84 and RV5. This high exhaustion values are also reflected in the good leveling properties for these dyes compared with those of RR84 and RV5.

### 3.3. Fastness Properties

As shown in Table 5, the fastness tests of washing, rubbing, and perspiration of samples that had been dyed with the reactive dyes are excellent to good indicating the existence of strong bonds (ionic and covalent bonds) between the dye molecules and the amidoximated acrylic fabrics. However, the wet rubbing fastness of dye RR4 and RR84 is fair. This result may be explained by the aggregation and/or surface coloration of these dyes.

## 4. Conclusion

Amidoximated acrylic fabrics could be dyed successfully with high fixation at acidic pH using nucleophilic addition type reactive dyes. Excellent to good fastness and levelling properties were obtained. Compared with substitution type reactive dyes, the results confirm the suitability nucleophilic addition type reactive dyes, which have small molecular sizes for the colouration of amidoximated acrylic fabrics. This finding is expected to pave the way for industry related textile colouration of acrylic fabrics and its blends with a variety of colours and dye classes.

## Figures and Tables

**Table 1:** Nitrogen content, tensile strength, and elongation at break of blank and modified acrylic fabrics.

Concentration of hydroxylamine hydrochloride, g/L	Nitrogen content %	Tensile strength Kg f	Elongation at break %
Blank	18.50	51.83	28.31
2	18.76	51.54	29.20
4	18.82	50.15	29.89
6	18.91	47.78	31.66
8	18.93	46.00	32.88
10	18.96	44.75	33.09
12	18.98	42.26	27.96
14	19.10	40.95	27.00

**Table 2:** FT-IR characteristic absorption peaks of modified acrylic fibers, RB19, and modified acrylic fabrics dyed with RB19.

Peak assignment	Wavenumber (cm^−1^)		
	Modified acrylic fibers	RB19	Modified acrylic fibers dyed with RB19
N–O stretching, amidoxime	947	—	930
C=N stretching, amidoxime	1594	—	1575
OH and NH_2_ stretching, amidoxime	3150–3550, broad	—	Present with less intensity
CN stretching, nitrile	2243	—	2243
S=O stretching, suffonic group	—	1040, 1140, 1228	Present overlapped with less intensity at 1228
C=O, C=C, conjugated carbonyl with aromatic	—	1500–1670	Present overlapped with less intensity at 1659

**Table 3:** FT-IR characteristic absorption peaks of modified acrylic fibers, RO16, and modified acrylic fabrics dyed with RO16.

Peak assignment	Wavenumber (cm^−1^)		
	Modified acrylic fibers	RO16	Modified acrylic fibers dyed with RO16
N–O stretching, amidoxime	947	—	968
C=N stretching, amidoxime	1594	—	1596
OH and NH_2_ stretching, amidoxime	3150–3550, broad	—	Present with more intensity
CN stretching, nitrile	2243	—	2243
S=O stretching, sulfonic group	—	1049, 1136, 1288	Present overlapped with less intensity at 1238
Para-disubstituted benzene	—	836	Present with less intensity at 828

**Table 4:** FT-IR characteristic absorption peaks of modified acrylic fibers, RR84, and modified acrylic fabrics dyed with RR84.

Peak assignment	Wavenumber (cm^−1^)		
	Modified acrylic fibers	RR84	Modified acrylic fibers dyed with RR84
N–O stretching, amidoxime	947	—	926
C=N stretching, amidoxime	1594	—	1632 overlapped with C=C aromatic and C=O amide
OH and NH_2_ stretching, amidoxime	3150–3550, broad	—	Present with more intensity
CN stretching, nitrile	2243	—	2243
C=C aromatic, C=O amide	—	1585, 1626, 1662	Overlapped with C=N amidoxime with less intensity at 1632
C–Br stretching	—	831	Present with less intensity at 848

**Table 5:** Fastness properties of dyed modified acrylic fabrics.

Reactive dyes^a^	Wash Fastness				Acid perspiration				Alkali perspiration				Rubbing fastness	
	St.∗	St.∗∗	St.∗∗∗	Alt.	St.∗	St.∗∗	St.∗∗∗	Alt.	St.∗	St.∗∗	St.∗∗∗	Alt.	Dry	Wet
RO16	4-5	4-5	4-5	4-5	4	4-5	4-5	4-5	4	4-5	4-5	4-5	3-4	3
RV5	4	4-5	4-5	4-5	4-5	4-5	4-5	4-5	4	4-5	4-5	4-5	4-5	3
RB19	4	4	4-5	4-5	4	4-5	4-5	4-5	4	4-5	4-5	4-5	4-5	3-4
RR84	4-5	4-5	4-5	4-5	4-5	4-5	4-5	4-5	4	4-5	4-5	4-5	3	2-3
RR4	4-5	4-5	4-5	4-5	4-5	4-5	4-5	4-5	4-5	4-5	4-5	4-5	3	2-3
RB171	4-5	4-5	4-5	4-5	4-5	4-5	4-5	4-5	4-5	4-5	4-5	4-5	3-4	3
RR195	4-5	4-5	4-5	4-5	4-5	4-5	4-5	4-5	4-5	4-5	4-5	4-5	4	3

∗St. = staining on cotton. ∗∗St. = staining on wool. ∗∗∗St. = staining on acrylic. Alt. = alteration.

^a^Reactive dyeing condition: shade 2% owf, LR 40 : 1, pH 2.5, at 100°C for 60 min.
